# The Association of Neighborhood Socioeconomic Status with Birth Weight is Not Confounded by Ambient Air Pollution in Twin Pregnancies: A Retrospective Cohort Study

**DOI:** 10.1159/neo/adsag016

**Published:** 2026-09-29

**Authors:** Marij Gielen, Esmée Bijnens, Eline Meireson, Steven Weyers, Catherine Derom, Tim Nawrot, Maurice P. Zeegers

**Affiliations:** a Department of Epidemiology, NUTRIM School for Translational Research in Metabolism, Maastricht University, Maastricht, The Netherlands; b Department of Environmental Sciences, Faculty of Science, Open Universiteit Heerlen, Heerlen, The Netherlands; c Centre for Environmental Sciences, Hasselt University, Diepenbeek, Belgium; d Department of Human Structure and Repair, University Ghent, Ghent, Belgium; e Department of Public Health and Primary Care, Leuven University, Leuven, Belgium

**Keywords:** Birth weight, Gestational age, Neighborhood income, Nitrogen dioxide, Particulate matter, Socioeconomic status, Twin

## Abstract

**Introduction:**

This study examines the association of neighborhood socioeconomic status (SES) with birth weight and gestational age in a population-based twin cohort, adjusting for ambient PM_10_ and NO_2_ exposure. By accounting for placentation, we aim to disentangle the independent and combined effects of socioeconomic and environmental factors.

**Methods:**

Neighborhood SES was defined using maternal residence at birth and statistical sectors (average area: 1.55 km^2^) to define annual median neighborhood household income. PM_10_ and NO_2_ exposure was estimated using a spatial-temporal interpolation method (Kriging) integrating daily monitoring data and land cover data. Analyses were stratified by placentation: one placental mass or two separate placentas. Multivariable linear mixed models were conducted for birth weight as outcome, linear regression models for gestational age.

**Results:**

For twins with two separate placentas, per EUR 3,000 (∼1 SD) increase in median neighborhood household income, birth weight increased with 46.83 grams. Adjustment for PM_10_ and NO_2_ did not change the associations. A part of these associations was explained by gestational age, as the association was reduced to 21.92 grams per EUR 3,000 increase. Each EUR 3,000 increase in annual median neighborhood household income corresponded to a longer gestation of 26 h, independent of ambient air pollution or other covariates.

**Discussion:**

Although neighborhood SES and ambient air pollution are correlated with birth outcomes, they act through different paths in this twin cohort. This approach provides insights into the etiology of adverse birth outcomes in a high-risk population, even in high-income settings with universal healthcare access.

## Introduction

Even in high-income countries, socioeconomic disparities contribute substantially to differences in morbidity and mortality [1]. These inequalities emerge early, with adverse birth outcomes disproportionately affecting socioeconomically disadvantaged populations. Socioeconomic status (SES) influences birth outcomes at both individual (e.g., maternal income, education) and contextual (e.g., neighborhood characteristics) levels. Neighborhood SES is independently associated with increased risks of preterm birth, small for gestational age (SGA), and low birth weight, even after adjustment for individual SES [2, 3]. This indicates that neighborhood environments exert distinct effects beyond individual characteristics.

Neighborhood income is a practical, spatially defined neighborhood SES indicator capturing broader physical and social conditions and is consistently associated with adverse perinatal outcomes [4]. Lower neighborhood income is linked to reduced healthcare access, poorer housing, greater exposure to stressors, and fewer opportunities for social support and healthy behaviors. These conditions may influence birth outcomes through chronic stress, inadequate prenatal care, poor maternal nutrition, and increased environmental burden [4–7]. These proxies may not adequately capture neighborhood deprivation in European high-income countries.

Air pollution is a key environmental risk factor for birth outcomes. Prenatal exposure to particulate matter (PM_10_) and nitrogen dioxide (NO_2_) is associated with reduced birth weight and increased preterm birth risk [8]. Importantly, even in high-income countries with relatively low ambient air pollution levels, negative effects remain detectable [8, 9].

Environmental exposures are also unequally distributed across socioeconomic strata [5, 10]. Socioeconomically disadvantaged populations are disproportionately exposed to air pollution, often living near traffic or industrial sources [11]. Consequently, SES and air pollution are interrelated, complicating estimation of their independent effects. Despite this overlap, relatively few studies have examined the joint effects of neighborhood SES and air pollution on birth weight. This is a critical gap, as omission of either factor may bias conclusions [6]. Some evidence suggests increased susceptibility to pollution among low-SES populations, amplifying risk [6, 7]. Studies adjusting for SES without accounting for pollution may misestimate associations, while those including both exposures are often US based and rely on proxies such as maternal education, race/ethnicity, or insurance status [10].

However, studies examining interactions between neighborhood SES and air pollution in relation to birth weight remain scarce in Europe. This gap is greater for twin pregnancies, which are inherently high risk, with shorter gestations and lower birth weights than singletons [12]. Placentation further modifies outcomes; twins sharing one placental (monochorionic and dichorionic) mass have lower birth weights and higher prematurity risk than those with two separate (dichorionic) placentas [13]. Given this vulnerability, socioeconomic and environmental stressors may have amplified effects, making twin pregnancies a relevant population to study combined exposures.

To date, research examining neighborhood SES and birth weight in twins while accounting for ambient air pollution remains limited. Previous analyses from the East Flanders Prospective Twin Survey (EFPTS) demonstrated trimester-specific effects of PM_10_ and NO_2_ on birth and placental weight [14–16]. Associations of pollutant exposure with birth outcomes were modified by placentation, emphasizing the need for stratified analyses in twin cohorts [14, 15]. However, the role of neighborhood SES income remains underexplored. Determining whether socioeconomic disadvantage independently affects birth weight after controlling for air pollution is essential for accurate risk estimation in European populations.

This study examines the association of neighborhood income, as a proxy of neighborhood SES, with birth weight and gestational age in a well-characterized twin cohort, adjusting for potential confounding by ambient PM_10_ and NO_2_ exposure. We hypothesize that neighborhood income is independently associated with these birth outcomes potentially ruling out the exposure to air pollution in socioeconomically disadvantaged populations, independent of traditional covariates. By accounting for placentation, we aim to isolate the independent and combined effects of neighborhood socioeconomic and environmental factors, providing insight into the etiology of adverse birth outcomes in a high-risk population and informing targeted public health interventions.

## Methods

### Study Population

This study utilized data from the EFPTS, a population-based registry of multiple births in East Flanders, Belgium [17], with a vast majority (>99%) of Caucasian origin. For this sub-study, maternal residences at birth were geocoded for 5,468 live born twins (=2,734 twin pairs) born between 1988 and 2013. Twin pairs of which one or both had a severe congenital malformation, unknown zygosity, median household income, parity, and unrealistic placental weight were excluded. Exposure to NO_2_ and PM_10_ was available for twins born after 2002 leading to a total study population of 4,660 twins (2,340 twin pairs) of which 138 twins (69 pairs) had missing information on the number of placentas resulting in a study sample of 4,542 live born twins (2,271 pairs) (Fig. 1).

**Fig. 1.**
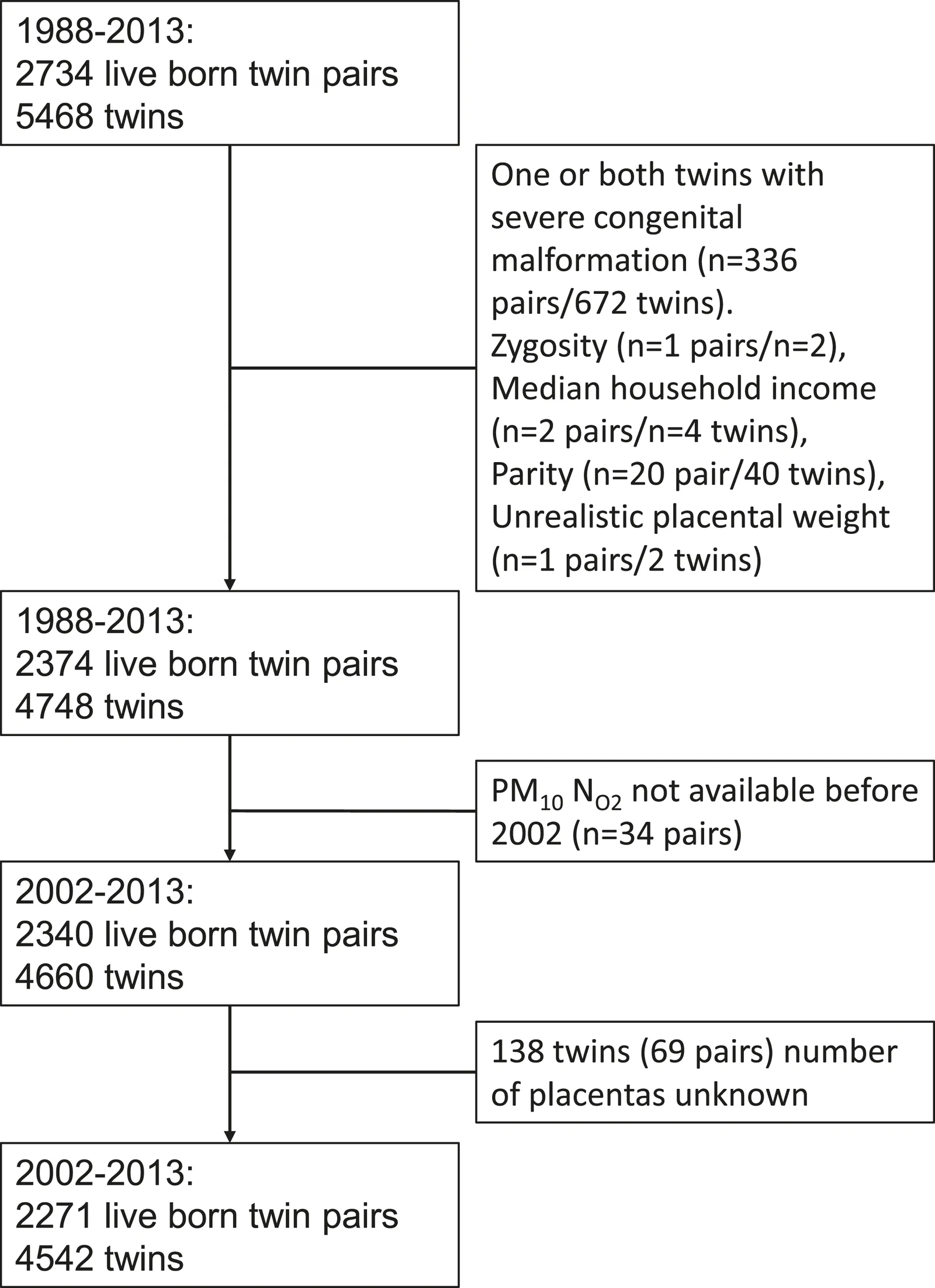
Flowchart.

### Data Collection

Obstetric and perinatal data were recorded, including year of birth, gestational age, maternal age, birth order, parity, sex, and neonatal survival [17, 18]. Placentas were examined within 24 h after delivery. The number of placentas was determined and the zygosity and chorionicity had an accuracy of 99% [19]. Informed consent was obtained from the parents at birth for registry data. Ethical approval, including the registry data from 2001 to 2013, was renewed based on current Belgian and European law by the Ghent University Hospital (METC: BC-04342) on June 25, 2020.

### Outcome

Primary outcome was birth weight (grams) and gestational age (weeks). Secondary outcomes included SGA, which is defined as birth weight below the 10th percentile of this dataset and preterm birth defined using international cutoff values. Gestational age (weeks) was based on the last menstruation or a first trimester ultrasound and was calculated as the number of completed weeks of pregnancy. Very preterm birth (birth before the 32nd week of gestation), preterm birth (born between 32 and 36 weeks), term birth (after 36 weeks) were also dichotomized into: preterm (very preterm and preterm birth) vs. term (≤36 weeks vs. >36 weeks).

### Exposure Neighborhood Income

Maternal residences were assigned to statistical sectors (average area = 1.55 km^2^), the smallest administrative entity of the Belgian National Institute of Statistics (NIS). Belgian census data (FOD Economie/DG Statistiek) derived from the NIS were used to define neighborhood SES based on annual household income in 2005.

### Environmental Confounders (PM_10_ and NO_2_)

Exposure to PM_10_ (µg/m^3^) and NO_2_ (µg/m^3^) was estimated using a spatial-temporal interpolation method (Kriging) based on daily measurements of monitoring stations (19 for PM_10_, 44 for NO_2_ in 2002, increasing to 36 and 64, respectively, in 2013) and land cover data (CORINE data set from European Environmental Agency) [20]. This model provided daily concentrations from 2002 to 2013 for 4 × 4-km^2^ grids from the Belgian telemetric air quality networks. Individuals’ mean exposure concentrations at the maternal residential address at time of delivery were estimated for specific periods (the overall pregnancy, the three trimesters, the last month, and the last week of pregnancy). PM_2.5_ exposure data were excluded due to limited availability and concerns regarding measurement stability [15].

### Traditional Covariates

Covariates included twin’s birth year and birth season (winter, spring, summer, fall), maternal age, sex of the twins, parity (primiparity vs. multiparity), zygosity-chorionicity group (dizygotic, monozygotic-dichorionic, and monozygotic-monochorionic), mode of conception (spontaneous, artificial ovulation, assisted reproductive technology, unknown), and number of placentas (one placental mass or two separate placentas). In case birth weight is the outcome, gestational age and birth order were additionally selected.

### Statistical Analysis

A conceptual diagram and detailed information about statistical analysis can be found in online supplementary material (for all online suppl. material, see https://doi.org/10.1159/neo/adsag016): conceptual diagram. Continuous variables are presented as mean (SD) or median (IQR) and proportions as number (*n*; %). Given a significant interaction between neighborhood income and number of placentas, we stratified into two groups: twins with one placental mass and twins with two separate placentas [14, 15].

For birth weight, twins were analyzed as individuals in a linear mixed model with a random intercept. For gestational age twins were analyzed as pairs in linear regression models.

Six exposure windows were analyzed: the whole pregnancy, the three trimesters, the last month, and the last week. The main analysis proceeded in four steps:

Univariable analysis was conducted between neighborhood income, the confounders PM_10_ or NO_2_ and outcomes.
Two-variable models: one of the environmental confounders (PM_10_ or NO_2_) was added to the univariable income model.
Third, both PM_10_ and NO_2_ were added to the univariable income model.
Multivariable analysis: income, PM_10_, and NO_2_ were added and adjusted for covariates (birth year of the twins, birth season, maternal age, sex of the twins, parity, zygosity-chorionicity-group, mode of conception). For birth weight, also gestational age and birth order were selected as covariates.

As gestational age is likely to be part of the causal pathway between neighborhood income and birth weight, sensitivity analysis without adjusting for gestational age was performed. Next, sensitivity analyses focused on SGA and preterm birth. Models and procedures mirrored those used for continuous outcomes but applied appropriate logistic regression techniques for binary outcomes: generalized mixed model (SGA) and logistic regression model (preterm birth).

## Results

The characteristics of the twins are presented in Table 1. Twins with one placental mass had lower birth weights and were more often SGA, had shorter gestational ages and were more often born preterm, had younger mothers who were more often primipara as compared to twins with two separate placentas. Twins with separate placentas were dichorionic, and more often opposite sex pairs and the result of infertility treatment.

**Table 1.** Baseline characteristics of the 4,542 twins (2,271 twin pairs) of EFPTS

​	All twins, *n* = 4,542 twins	Twins with one placental mass*, *n* = 2,634 twins	Twins with 2 separate placentas, *n* = 1,908 twins	*p* value**
	mean (SD) median (IQR) *n* (%)	mean (SD) median (IQR) *n* (%)	mean (SD) median (IQR) *n* (%)	
Zygosity and chorionicity	​	​	​	<0.001
DZDC	3,336 (73.5)	1,644 (62.4)	1,692 (88.7)	​
MZDC	370 (8.2)	154 (5.9)	216 (11.3)	​
MZMC	836 (18.4)	836 (31.7)	0	​
Birth weight (gram)	2,434 (521)	2,381 (520)	2,508 (513)	<0.001
SGA	​	​	​	0.002
<10th percentile of this group	1,145 (25.2)	710 (27.0)	435 (22.8)	​
≥10th percentile of this group	3,397 (74.8)	1,924 (73.0)	1,473 (77.2)	​
Sex	​	​	​	0.001
Male	2,247 (49.5)	1,249 (47.4)	998 (52.39)	​
Female	2,295 (50.5)	1,385 (52.6)	910 (47.7)	​
Sex of the twin pair (pairs)	​	​	​	<0.0001
Male-male	714 (31.4)	432 (32.8)	282 (29.6)	​
Male-female	819 (36.1)	385 (29.2)	434 (45.5)	​
Female-female	738 (32.5)	500 (38.0)	238 (24.9)	​
Gestational age (weeks)	36 (35–37)	36 (35–37)	36 (35–37)	<0.001
	35.8 (2.3)	35.6 (2.4)	36.0 (2.2)	<0.001
Preterm birth	​	​	​	<0.001
<32 weeks	274 (6.0)	186 (7.1)	88 (4.6)	​
≥32 and ≤36 weeks	2,266 (49.9)	1,368 (51.9)	898 (47.1)	​
>36 weeks	2,002 (44.1)	1,080 (41.0)	922(48.3)	​
Parity (= primipara or higher)	​	​	​	0.002
Primiparity	2,358 (51.9)	1,420 (53.9)	938 (49.2)	​
Multiparity	2,184 (48.1)	1,214 (46.1)	970 (50.8)	​
Maternal age (years)	30.27 (4.6)	30.12 (4.50)	30.47 (4.67)	0.01
Birth season	​	​	​	0.76
Fall	1,106 (24.3)	634 (24.1)	472 (24.7)	​
Spring	1,098 (24.2)	648 (24.6)	450 (23.6)	​
Summer	1,190 (26.2)	680 (25.8)	510 (26.7)	​
Winter	1,148 (25.3)	672 (25.5)	302 (25.0)	​
Mode of conception	​	​	​	<0.001
Spontaneous conception	2,612 (57.5)	1,608 (61.1)	1,004 (52.6)	​
Artificial ovulation induction only	658 (14.5)	352 (13.4)	306 (16.0)	​
Assisted Reproductive Technology	1,150 (25.3)	596 (22.6)	554 (29.0)	​
Unknown mode of conception	122 (2.7)	78 (3.0)	44 (2.3)	​

For continuous variables and with a normal distribution, data are given as means and standard deviations and for non-normally distributed data as median and interquartile range. For categorical variables, data are given as frequencies and percentages.

DZ, dizygotic; MZ, monozygotic; DC, dichorionic; MC, monochorionic.

*2 fused placentas (MZDC or DZDC) or one placenta (MZMC twins).

**for continuous variables with a normal distribution, Student’s *t* test; with a non-normal distribution, Wilcoxon two-sample test; and for categorical variables, chi-square test were performed.

There were no differences in annual median neighborhood income between twins with one placental mass and twins with two separate placentas. PM_10_ µg/m^3^ and NO_2_ were higher for twins with one placental mass during the last month and last week as compared to twins with two separate placentas. Concentrations of NO_2_ were higher for the whole pregnancy for twins with one placental mass (Table 2, online suppl. Table 1).

**Table 2.** Maternal exposure characteristics of the 2,271 twin pairs of EFPTS

​	All twins, *n* = 2,271 twin pairs	Twins with one placental mass*, *n* = 1,317 twin pairs	Twins with 2 separate placentas, *n* = 954 twin pairs	*p* value**
Neighborhood annual median income €	19,895 (3,140)	19,896 (3,172)	19,894 (3,097)	0.99
PM_10_, µg/m^3^				
Trimester 1	30.9 (6.0)	30.9 (5.9)	30.8 (6.1)	0.62
Trimester 2	30.8 (6.3)	30.8 (6.3)	30.7 (6.3)	0.43
Trimester 3	30.3 (7.1)	30.4 (7.3)	30.0 (6.8)	0.08
Last month	30.2 (8.0)	30.4 (8.1)	29.9(7.8)	0.03
Last week	27.5 (21.9–36.2)	27.5 (21.8–36.6)	27.5 (22.0–35.0)	0.30
	30.1 (11.3)	30.5 (11.8)	29.7 (10.6)	0.01
Whole pregnancy	30.7 (4.6)	30.8 (4.6)	30.6 (4.7)	0.15
NO_2_, µg/m^3^				
Trimester 1	24.5 (7.1)	24.6 (7.1)	24.3 (7.2)	0.18
Trimester 2	24.5 (7.3)	24.6 (7.3)	24.2 (7.3)	0.06
Trimester 3	24.2 (7.7)	24.4 (7.7)	24.0 (7.6)	0.07
Last month	24.1 (8.0)	24.3 (8.1))	23.8 (7.8)	0.02
Last week	22.8 (17.2–29.6)	23.11 (17.5–29.9)	22.4 (16.8–28.8)	0.03
	24.0 (9.4)	24.2 (9.4)	23.7 (9.2)	0.05
Whole pregnancy	24.4 (5.9)	24.6 (5.8)	24.2 (5.9)	0.03

For continuous variables and with a normal distribution, data are given as means and standard deviations and for non-normally distributed data, as median and interquartile range. For categorical variables data are given as frequencies and percentages.

*2 fused placentas (MZDC or DZDC) or one placenta (MZMC twins).

**for continuous variables with a normal distribution, Student’s *t* test; with a non-normal distribution, Wilcoxon two sample test; and for categorical variables, chi-square test were performed.

The main analysis revealed a significant association of SES with outcomes for twins with two separate placentas. Per EUR 1,000 increase of annual median neighborhood household income, birth weight increased by 15.61 grams. Accordingly, a EUR 3,000 increase (∼1 SD) was associated with 46.83 grams increase in birth weight. After adjusting for PM_10_ and NO_2_ the associations of median neighborhood household income with birth weight remained the same without meaningful differences between all six exposure windows (Table 3, online suppl. Table 2) indicating that PM_10_ and NO_2_ did not explain the SES effect.

**Table 3.** Association between annual neighborhood income and birth weight or gestational age: adjusted analyses for twins with two separate placentas

	Birth weight two separate placentas (1,908 twins)			Gestational age two separate placentas (954 twin pairs)		
	beta SES^1^	95% CI	*p* value	beta SES^1^	95% CI	*p* value
Unadjusted						
	15.609	5.886–25.332	0.017	0.052	0.006–0.097	0.025
Adjusted for PM_10_						
Trimester 1	15.461	5.724–25.199	0.002	0.052	0.006–0.097	0.026
Trimester 2	15.501	5.758–25.244	0.002	0.052	0.006–0.097	0.026
Trimester 3	14.944	5.462–24.425	0.002	0.051	0.008–0.094	0.021
Last week	16.586	6.823–26.349	0.001	0.056	0.010–0.101	0.017
Last month	16.234	6.457–26.011	0.001	0.056	0.011–0.103	0.014
Whole pregnancy	15.426	5.659–25.192	0.002	0.052	0.007–0.098	0.025
Adjusted for NO_2_						
Trimester 1	15.356	5.487–25.225	0.002	0.053	0.007–0.099	0.025
Trimester 2	15.759	5.858–25.661	0.002	0.055	0.009–0.101	0.020
Trimester 3	16.193	6.555–25.831	0.001	0.058	0.014–0.102	0.009
Last week	16.931	7.038–26.823	0.001	0.059	0.012–0.105	0.013
Last month	16.970	7.040–26.899	0.001	0.063	0.016–0.109	0.008
Whole pregnancy	15.893	5.901–25.885	0.002	0.057	0.011–0.104	0.016
Adjusted for PM_10_ and NO_2_						
Trimester 1	15.647	5.725–25.569	0.002	0.054	0.008–0.101	0.022
Trimester 2	16.155	6.191–26.119	0.002	0.057	0.011–0.104	0.016
Trimester 3	15.510	5.873–25.148	0.002	0.054	0.010–0.098	0.016
Last week	16.691	6.794–26.587	0.001	0.058	0.012–0.104	0.014
Last month	16.879	6.934–26.824	0.001	0.062	0.015–0.108	0.009
Whole pregnancy	16.195	6.169–26.220	0.002	0.058	0.012–0.105	0.015
Multivariable analysis full model^2^						
Trimester 1	6.819	0.513–13.125	0.034	0.057	0.010–0.104	0.018
Trimester 2	6.984	0.666–13.303	0.030	0.056	0.009–0.104	0.019
Trimester 3	6.872	0.512–13.232	0.034	0.056	0.012–0.101	0.013
Last week	7.666	1.380–13.953	0.017	0.057	0.010–0.104	0.017
Last month	7.039	0.687–13.390	0.030	0.063	0.016–0.110	0.009
Whole pregnancy	6.821	0.475–13.168	0.035	0.059	0.012–0.106	0.014
Multivariable analysis backward^3^						
Trimester 1	7.474	1.299–13.657	0.018	0.054	0.007–0.100	0.024
Trimester 2	7.515	1.301–13.729	0.018	0.057	0.010–0.103	0.018
Trimester 3	7.275	1.045–13.505	0.022	0.053	0.009–0.098	0.018
Last week	7.834	1.655–14.012	0.013	0.058	0.011–0.104	0.015
Last month	7.337	1.124–13.551	0.021	0.062	0.015–0.109	0.010
Whole pregnancy	7.306	1.056–13.557	0.022	0.058	0.011–0.105	0.016

Gestational age adjusted for: one placental mass – zygosity-chorionicity group, parity, birth year, and mode of conception; two separate placentas – zygosity-chorionicity group, parity, maternal age, birth year, and mode of conception.

gestational age adjusted for: zygosity-chorionicity group, sex of the twin, parity, maternal age, birth year, birth season, mode of conception.

^1^ SES, the unit of annual median neighborhood income is given by EUR 1,000, indicating that per EUR 1,000 increase of income, birth weight increased by, for example, 15.61 grams and gestational age by 0.052 weeks in unadjusted analysis.

^2^ Full-model birth weight adjusted for: zygosity-chorionicity group, sex of the twin, parity, gestational age, birth order, maternal age, birth year, birth season, mode of conception.

^3^ Birth weight adjusted for: one placental mass – zygosity-chorionicity group, sex of the twin, parity, gestational age, birth order; two separate placentas – sex of the twin, parity, maternal age, gestational age, birth order. Gestational age adjusted for: one placental mass – zygosity-chorionicity group, parity, birth year, and mode of conception; two separate placentas – zygosity-chorionicity group, parity, maternal age, birth year, and mode of conception.

Multivariable analysis showed that the estimated association of median neighborhood household income with birth weight attenuated, with no meaningful differences between the fully adjusted model and the model with backward elimination (Table 3). For example, for the whole pregnancy, the estimate decreased from 15.426 to 7.306. Per EUR 1,000 increase of median neighborhood income, birth weight increased with 7.3 gram. Per EUR 3,000 (∼1 SD), birth weight increased with 21.92 gram. Without adjustment for gestational age, the estimate did not change substantially (beta 15.941) (online suppl. Table 5), indicating that gestational age is partly a mediator and confounder in the association of SES with birth weight.

A similar linear relationship was observed for gestational age. Per EUR 1,000 increase of annual median neighborhood household income, gestational age increased with 0.052 weeks (∼8.7 h), which cannot be explained by ambient air pollution. Per EUR 3,000 (∼1 SD) increase in annual median neighborhood household income, gestational age increased with 0.156 weeks (∼26 h). After adjustment for PM_10_ and NO_2_, the associations between median neighborhood household income and birth weight remained largely unchanged and results remained robust in the multivariable analysis (Table 3, online suppl. Table 3).

No significant associations were found for twins with one placental mass. Per EUR 1,000 increase of annual median neighborhood household income, birth weight decreased by 1.4 gram (95% CI −9.6–6.8). Formerly, no further analysis was required. For reasons of completeness, these results are shown in the online supplementary Tables 1–6.

Univariable analysis with SGA or preterm birth as outcome revealed that median income was not associated with the outcome, and no further analysis was required. For twins with two separate placentas, the OR for SGA was 0.97 (95% CI 0.92–1.03) and OR for preterm birth of 0.99 (95% CI 0.95–1.03) per EUR 1,000 increase of annual median neighborhood household income. For twins with one placental mass, the OR for SGA was 0.97 (95% CI 0.93–1.01) and OR for preterm birth of 1.00 (95% CI 0.97–1.04). For reasons of completeness, these results are shown in the online supplementary Tables 4, 7–10.

## Discussion

This study investigated the association of neighborhood SES with birth weight and gestational age in a well-characterized twin cohort, while adjusting for confounding by PM_10_ and NO_2_ exposure and accounting for placentation. Neighborhood household income is positively associated with birth weight and gestation in twins with two separate placentas (dichorionic), but not in those with one placental mass. Specifically, each EUR 3,000 increase in annual median neighborhood income corresponded to a 47-gram higher birth weight and 26-h longer gestation. After adjusting for PM_10_ and NO_2_ exposure, the association remained largely unchanged without meaningful differences between the six exposure windows. After multivariable adjustment, the association reduced to 22 gram per EUR 3,000, while after multivariable adjustment without gestational age, the association with birth weight remained; the association with gestational age persisted.

We confirmed a statistically significant association of neighborhood SES with both outcomes in dichorionic twins. Because air pollution was explicitly modeled, the estimates are unlikely to be inflated by confounding. Although a 47-g increase may appear modest, a 94-g difference across two standard deviations of neighborhood income is comparable to established determinants of twin birth weight, including sex (∼105 g), parity (∼118 g), and mode of conception (∼34 g). This supports clinical relevance. While our study’s air pollutant levels were above the WHO air pollution guidelines but were comparable to or slightly lower than in other European studies from similar time periods, pollutant effects on birth outcomes are stronger than those reported in singletons [15]. Persistence of SES effects after pollution adjustment suggests no evidence of meaningful confounding of air pollution within the observed exposure range within this cohort. Neighborhood income and air pollution may be operating through largely independent pathways. Gestational age likely lies on the causal pathway between SES and birth weight and may partially mediate this association, which could explain the attenuation after adjustment. Consequently, not adjusting for gestational age may overestimate the association, whereas adjusting for it may underestimate the total effect. Another explanation could be the limited shared variance between the exposures within the observed exposure range in addition to the ethnically homogeneous population.

We only found an association for twins with two separate placentas. Twins with one placental mass represent two distinct groups: twins with two separate fused placentas (dizygotic and monozygotic-dichorionic) and twins with one shared placenta (monozygotic-monochorionic). This heterogeneity, which also introduces differences in biological and physiological characteristics, may dilute possible associations. Zygosity and chorionicity do influence placental weight in twins with one placental mass [13]. Monozygotic-monochorionic twins have lower birth weights and smaller placentas [21]. Moreover, monochorionic twins are associated with higher risks of prematurity, low birth weight, and birth weight discordance due to unequal placental sharing [21]. These intrinsic risks are likely to obscure or reduce socioeconomic effects. Additionally, air pollution showed stronger associations with outcomes in this group, suggesting that environmental stressors may dominate. In this context, environmental toxicants may override SES-related influences.

Despite increased susceptibility in pregnancy [9, 22] and evidence linking prenatal air pollution exposure to lower birth weight [16, 23–25], we found no interaction between neighborhood income and pollution. Although lower SES populations are more likely to reside in polluted areas [6], there was no statistical evidence of effect modification (online suppl. Table 11), although exposure misclassification in air pollution estimates may attenuate the ability to detect confounding or interaction. Both SES-related stressors and pollution may operate through overlapping biological pathways [26–28]; the absence of interaction suggests that SES and air pollution act independently in this cohort.

SES is a multidimensional construct encompassing income, education, occupation, and contextual factors. We used neighborhood household income as a proxy for SES. While imperfect, it captures broader structural and environmental conditions relevant to maternal and fetal health, although in this study, we could not distinguish whether the observed associations reflect contextual neighborhood effects, individual-level socioeconomic factors, or a combination of both. Lower neighborhood income is associated with adverse maternal conditions, including poor nutrition, psychosocial stress, limited prenatal care, and higher environmental exposure. In twin pregnancies, already characterized by elevated baseline risks, these factors may compound adverse outcomes. Individual SES indicators do not always correlate strongly and neighborhood SES has independent effects beyond individual characteristics [29].

This study has several strengths. It is based on a large, prospective, population-based twin cohort, reducing selection bias and enabling stratified analyses. Detailed placental data (zygosity, chorionicity) allowed biologically informed interpretation. Air pollution exposure was estimated using residential address-based models.

Limitations must be acknowledged. First, potential exposure misclassification is possible. Exposure misclassification of pollution is possible due to limited spatial resolution and reliance on residential address at delivery, without accounting for mobility, workplace exposure, or commuting and exposure misclassification in the assignment neighborhood income since a single cross-sectional indicator was used. Temporal mismatch may exist because neighborhood income can change over time. However, stable spatial patterns of socioeconomic disadvantage suggest the measure captures enduring neighborhood SES differences rather than annual fluctuations [30, 31]. These limitations likely led to non-differential misclassification and attenuation of effects. Second, neighborhood income does not capture individual-level SES or health behaviors, potentially leaving residual confounding. Third, key individual confounders such as smoking, BMI, nutrition, physical activity, stress, and pregnancy complications were unavailable. Smoking and alcohol use are strongly patterned by SES and may mediate associations. Prior research suggests SES effects may operate through maternal BMI and gestational weight gain [3]. Fourth, the ethnically homogeneous population (>99% Caucasian) limits generalizability. Fifth, mediation pathways were not formally assessed nor were long-term outcomes.

## Conclusion

This study supports a modest but robust association of neighborhood income with birth weight in twins with two separate placentas, independent of air pollution. No associations were observed in twins with a single placental mass, likely due to biological masking effects related to monochorionic placentation and greater vulnerability to air pollution.

These findings highlight the importance of neighborhood-level conditions for fetal health, even in high-income settings. Further studies incorporating individual-level SES, behavioral factors, and long-term follow-up are warranted to clarify underlying mechanisms of fetal and infant development.

## Acknowledgments

We are grateful for the families who participated in this study, without whom this study could not have been performed. ChatGPT was used to edit the text of the Introduction and Discussion sections. No AI was used in gathering references, data collection, analysis, and the writing of the Methods and Results sections of this manuscript.

## Statement of Ethics

This study was designed according to the Declaration of Helsinki. Ethical approval, including the registry data from 2001 to 2013, was renewed based on current Belgian and European law by the Ghent University Hospital (METC: BC-04342) on June 25, 2020. Written informed consent was obtained from the parents at birth for registry data.

## Conflict of Interest Statement

The authors declare that they have no known competing interests or personal relationships that could have appeared to influence the work reported in this paper.

## Funding Sources

This work was supported by the EU Research Council “project ENVIRONAGE” (ERC-2012-StG 310890), Flemish Scientific Fund (G087311N10 and G073315N). Since its origin, the East Flanders Prospective Twin Survey has been partly supported by grants from the Fund of Scientific Research, Flanders and Twins, a non-profit Association for Scientific Research in Multiple Births (Belgium). The funders had no role in the study design, data collection, data analysis, and manuscript conception, planning, writing, and decision to publish.

## Author Contributions

M.G.: conceptualization, data curation, formal Analysis, and writing – original draft, review, and editing. E.M.: investigation and writing – review and editing. S.W., C.D., and T.N.: investigation and resources. E.B.: investigation, resources, and writing – review and editing. M.Z.: conceptualization, supervision, and writing – review and editing.

## Data Availability

Restrictions apply to the availability of these data, which were used under license for the current study. Data are, however, available from the authors upon reasonable request and with permission of the board of the EFPTS. Further inquiries can be directed to the corresponding author.
